# Mapping the Fitness Landscape of Gene Expression Uncovers the Cause of Antagonism and Sign Epistasis between Adaptive Mutations

**DOI:** 10.1371/journal.pgen.1004149

**Published:** 2014-02-27

**Authors:** Hsin-Hung Chou, Nigel F. Delaney, Jeremy A. Draghi, Christopher J. Marx

**Affiliations:** 1 Department of Organismic and Evolutionary Biology, Harvard University, Cambridge, Massachusetts, United States of America; 2 Institute of Molecular Systems Biology, ETH Zurich, Zurich, Switzerland; 3 Department of Zoology, University of British Columbia, Vancouver, Canada; 4 Department of Biology, University of Pennsylvania, Philadelphia, Pennsylvania, United States of America; 5 Faculty of Arts and Sciences Center for Systems Biology, Harvard University, Cambridge, Massachusetts, United States of America; Warwick Medical School, United Kingdom

## Abstract

How do adapting populations navigate the tensions between the costs of gene expression and the benefits of gene products to optimize the levels of many genes at once? Here we combined independently-arising beneficial mutations that altered enzyme levels in the central metabolism of *Methylobacterium extorquens* to uncover the fitness landscape defined by gene expression levels. We found strong antagonism and sign epistasis between these beneficial mutations. Mutations with the largest individual benefit interacted the most antagonistically with other mutations, a trend we also uncovered through analyses of datasets from other model systems. However, these beneficial mutations interacted multiplicatively (i.e., no epistasis) at the level of enzyme expression. By generating a model that predicts fitness from enzyme levels we could explain the observed sign epistasis as a result of overshooting the optimum defined by a balance between enzyme catalysis benefits and fitness costs. Knowledge of the phenotypic landscape also illuminated that, although the fitness peak was phenotypically far from the ancestral state, it was not genetically distant. Single beneficial mutations jumped straight toward the global optimum rather than being constrained to change the expression phenotypes in the correlated fashion expected by the genetic architecture. Given that adaptation in nature often results from optimizing gene expression, these conclusions can be widely applicable to other organisms and selective conditions. Poor interactions between individually beneficial alleles affecting gene expression may thus compromise the benefit of sex during adaptation and promote genetic differentiation.

## Author Summary

The pace and outcome of a series of adaptive steps in an evolving lineage depends upon how well different beneficial mutations stack on top of each other. We found that independent beneficial mutations that affected gene expression for a metabolic pathway did not work well together, and were often jointly deleterious. The most beneficial mutations interacted the most poorly with others, which was a trend we found common in other biological systems. Through generating a model that accounted for enzymatic benefits and expression costs, we uncovered that this antagonism was caused by a phenotype to fitness mapping that had an intermediate peak. This allowed us to predict the fitness effect of double mutants and to uncover that the single winning mutations tended to move straight to the peak in a single step. These findings demonstrate the importance of considering the phenotypic changes that cause nonlinear interactions between mutations upon fitness, and thus influence how populations evolve.

## Introduction

The concept of a fitness landscape unites the three levels of evolutionary change – genotype, phenotype, and fitness – into a mathematical picture of the potential for, and constraints upon, adaptive evolution. By mapping genotypes to a measure of fitness, fitness landscapes guide our understanding of how epistasis – nonlinear interactions between the fitness effects of mutations – shapes evolution. Strong epistasis implies that landscapes are rugged, with many peaks, or locally optimally genotypes [1], [2]. The magnitude and form of epistasis is predicted to determine the number of evolutionary trajectories [3], [4], the rate and repeatability of adaptation [5]–[7], and the benefit of sex [8]. Recent experimental work with a wide variety of model organisms has revealed diminishing returns as a general trend of adaptation [9]–[13], with relatively few cases of synergy [11], [14] or sign epistasis [15] (i.e., the same mutation being beneficial or deleterious in different contexts [16]). Antagonism between adaptive mutations might imply that these populations are summiting peaks in their fitness landscapes with just a handful of genetic changes. This explanation might lead to further trends, such as a negative relationship between the initial selective coefficient of a mutation and its epistatic interactions that could prove to be a useful predictor of a saturating process of adaptation [17]. In order to definitely link diminishing returns to the ascent of local peaks, as well as to understand the existence of the peaks themselves, we must understand the phenotypes that link genotype and fitness in the adaptive landscape. Mathematically convenient formulations such as Fisher's geometric model for adaptation near a single peak [18] have been used to interpret the trend toward antagonism [19]. This approach assumes stabilizing selection a priori. What remains unclear is what types of physiological interactions give rise to fitness landscapes of varying shape and form, as well the constraints upon mutational changes to underlying phenotypes.

Models of metabolic pathways have been amongst the most successful in translating underlying biochemical phenotypes to fitness. The contribution of enzyme activities upon metabolic flux has been formalized via Metabolic Control Analysis (MCA) [20], [21]. The ability of this approach to predict the fitness consequences of changes in enzyme properties has been verified in experimental systems that vary from *Escherichia coli* in lactose-limited chemostats to the flight properties of butterflies (reviewed in [22]). Turning to multiple enzymes, MCA theory has suggested a general trend toward synergistic interactions between activity-increasing mutations in a metabolic pathway [23], [24]. A major limitation, however, has been that the costs of enzyme expression [25] have not been included in classical MCA. Whereas the dependence of flux through a metabolic pathway saturates with increasing levels of a given enzyme, the costs will continue to accumulate. The balance of these two selective factors will generate an intermediate optimum, and thus stabilizing selection. Inclusion of expression costs to MCA has enabled predictions of the optimum levels of a single enzyme [26], and was used to compare the differential utility of alternate, degenerate pathways [27]. An open question, however, is how the balance between catalytic benefits and expression costs plays out to optimize enzyme expression across many enzymes simultaneously.

**Figure 1:**
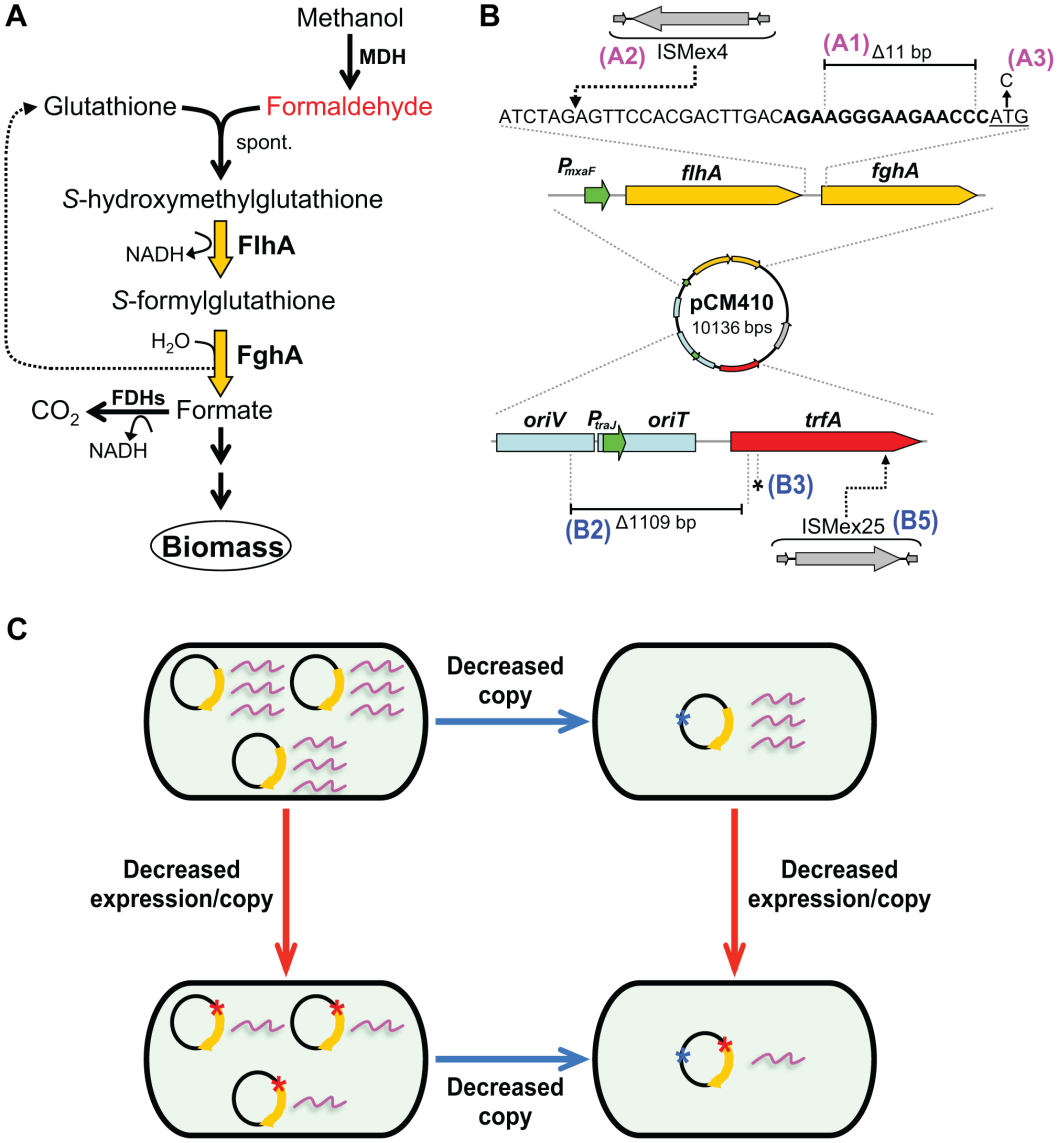
Adaptive mutations that optimized the expression of the GSH-linked pathway. A) The GSH-linked formaldehyde oxidation pathway in the EM strain. The enzymes of the GSH-linked pathway are indicated with yellow arrows. MDH, methanol dehydrogenase; spont., spontaneous reaction of formaldehyde and GSH; FlhA, *S*-hydroxymethyl GSH dehydrogenase; FghA, *S*-formyl-GSH hydrolase; FDHs, formate dehydrogenases. B) Adaptive mutations identified on the introduced plasmid (pCM410) expressing the introduced formaldehyde pathway. Mutations occurring in the predicted ribosome-binding site (bold text) of *fghA*, or its upstream region are shown in magenta (Class A). Mutations occurring in regions that control plasmid replication are shown in blue (Class B). The *fghA* start codon is underlined. *trfA*, a gene encoding the TrfA protein essential to plasmid replication; *P_mxaF_*, promoter of the *flhA*-*fghA* gene cassette; *oriV*, origin of replication recognized by the TrfA protein; *oriT*, origin of transfer. ISMex4 and ISMex25, two insertion sequences native to *M. extorquens* AM1. C) Diagram of orthogonal mechanisms of Class A and B mutations on gene expression.

In order to study how evolution would simultaneously optimize expression of multiple genes, we have developed a model system of an engineered *Methylobacterium extorquens* AM1 (EM) in which we altered its central metabolism to be dependent upon a foreign pathway (Figure 1A for details). *M. extorquens* grows on methanol by oxidizing it first to formaldehyde, and then through a series of steps to formate, which is either fully oxidized to CO_2_ or incorporated into biomass [28]–[32]. In the EM strain we removed the endogenous pathway for formaldehyde oxidation in wild-type (WT) [28] and replaced it with two genes encoding a foreign pathway that oxidizes formaldehyde via glutathione (GSH) derivatives [29]. Eight populations dependent upon this introduced metabolic pathway evolved in methanol-containing medium via serial transfers for 900 generations [10], [33].

Adaptation of the unfit EM strain to grow on methanol consistently involved beneficial mutations that altered expression of the foreign GSH pathway (Figure 1B). When the GSH pathway was introduced, the two enzymes were cloned together on a single mRNA transcript behind a strong native promoter present on a medium copy plasmid (∼9 cell^−1^) [10], [29], [34], [35]. As such, the costs of expression outweighed the catalytic benefits, and among the targets of adaptation we identified by resequencing strains evolved in separate populations, we universally obtained beneficial mutations that decreased expression of these enzymes [10], [34]. These mutations reduced expression of the GSH pathway through three classes of underlying mechanisms: Class A decreased expression per gene copy, Class B reduced gene dosage by lowering plasmid copy number, and Class C integrated the introduced pathway into the host genome, which also reduced plasmid copy number [33], [34] (Figure 1B). In terms of epistasis, mutations in multiple genes along a single adaptive trajectory – including one mutation (here ‘A1’) reducing expression of the GSH pathway – have been shown to exhibit a general trend of diminishing returns that was devoid of sign epistasis [10]. However, here we are interested in uncovering the trends and mechanisms underlying epistatic interactions between mutations that arose in separate adapting lineages and affect expression of the same metabolic pathway.

We combined independently-arising beneficial mutations affecting gene expression of this two-enzyme metabolic pathway and report strong antagonism and sign epistasis for fitness. These interactions were increasingly antagonistic for larger benefit mutations. Such strong antagonism did not stem from the effects of mutational combinations upon enzyme levels, but rather from the nonlinear mapping between enzyme expression and organismal fitness. By developing a quantitative model that relates expression cost and catalytic benefit to fitness, we characterized the overall shape of this fitness landscape and revealed that some of these single mutations can optimize multiple phenotypes simultaneously, leading to a big jump toward the single, global optimum.

## Results

### Interactions between mutations affecting expression of a two-enzyme metabolic pathway exhibit strong antagonism and sign epistasis

**Figure 2:**
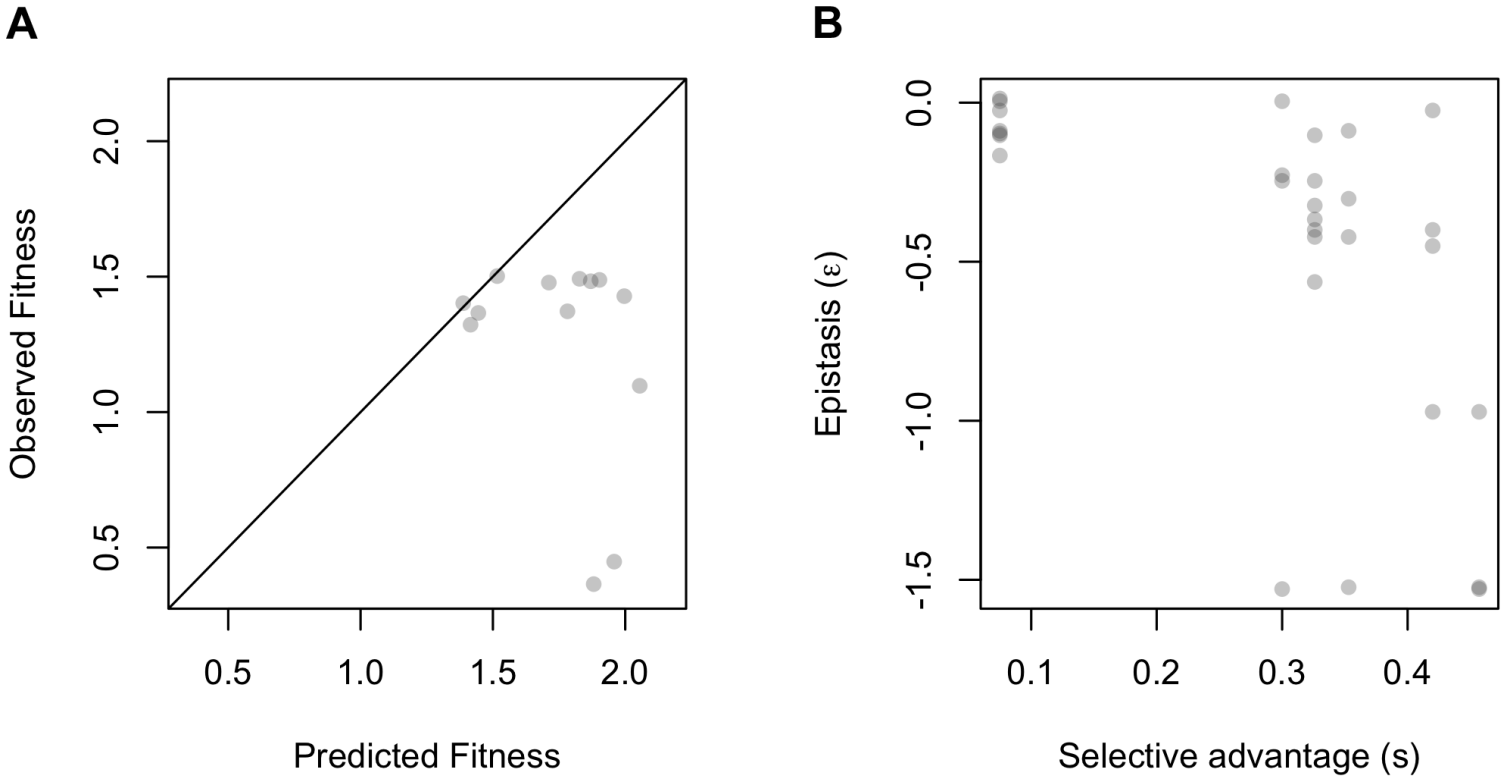
Epistasis at the level of expression phenotypes and fitness relative to independence or kinetic model. A) Fitness values of mutation combinations are consistently lower than expected by multiplicative independence. B) Mutations with larger with selective advantages (*s*) when tested alone in the ancestor have more negative ε values in combination with other mutations.

To explore the pattern of epistatic interactions between beneficial mutations affecting expression of the GSH-dependent pathway, we combined beneficial plasmid mutations that emerged during experimental evolution and affected distinct traits [34]. We focused upon Class A (decreased expression per copy) and B (reduced gene dosage) mutations because of their genetic tractability, and the prediction that these represent orthogonal mechanisms to achieve lower expression. We hypothesized that mutational combinations between these classes would result in enzyme levels that would be the product of the individual perturbations (Figure 1C). Three class A mutations, A1–A3, and one class B mutation, B5, occurred independently, whereas B2 and B3 were isolated together from the same plasmid. We generated 12 plasmids that paired each Class A mutation with each one from Class B, as well as with the B2–B3 pair, and measured their relative fitness via competitions with a fluorescently labeled ancestor [34] (Tables S1, S2). The observed fitness values for the mutational combinations were substantially less than expected based upon a simple multiplicative null model incorporating the single mutant effects (i.e., *W_ij_* = *W_i_*×*W_j_*; R^2^ = 0.53, adj-R^2^ = 0.32; Figure 2A).

The increasingly strong antagonism for higher expected fitness values suggested a potential negative relationship between the selective coefficients observed for each mutation and the average epistasis that mutation exhibited with other mutations. We observed that the individually most beneficial mutations (large *s*) engendered the greatest antagonism (ε<0) when combined with other mutations, including several examples of sign epistasis (Figure 2B).

### The observed relationship between selective coefficient and average epistasis is observed for other biological systems

Several theoretical arguments suggest that the geometry of fitness landscapes might induce correlations between the size of a mutation and the strength and direction of epistasis. Epistasis has been observed to be coupled to the mean fitness effect of mutations [36], [37]. A single beneficial mutation of large effect may appreciably change both the mean fitness of subsequent mutations and the remaining distance to the optimum, potentially skewing its own epistatic coefficients.

Given the emerging empirical consensus and theoretical arguments for antagonistic epistatic interactions among beneficial mutations, we analyzed several other datasets to ask whether the strength and form of the relationship between selective effect and average epistatic effect held for intragenic and intergenic datasets. For this comparison we analyzed the relationship between s and ε for previous datasets from *M. extorquens* and *E. coli* where the beneficial mutations occurred consecutively in a variety of genes across the genome of a single adapting lineage [10], [11], combinations of mutations from two genes of the bacteriophage ID11 [12], and two datasets of within-protein interactions for β-lactamase [17], [38]. These datasets also displayed signs of a correlation between *s* and increasingly negative ε (as noted in [37]), with the exception of the intragenic data for β-lactamase (Figure S1).

Negative trends in the relationship between initial selective coefficient and epistasis may seem like obvious evidence for diminishing returns. However, recent theoretical work has shown that in models where mutations have random effects and no tendency to be either synergistic or antagonistic, a pattern of diminishing returns occurs between mutations if they are selected conditioned on being beneficial in the ancestral background [39]. In Supplementary Text S1 we show this behavior in a simple model of evolution on fitness landscapes with no mean epistatic tendency and show how it leads to a pattern of diminishing returns between beneficial mutations as a form of regression to the mean. This analysis suggests that genotype-fitness data alone, without knowledge of the phenotypic effects of mutations or the physiological causes for trade-offs, might be insufficient to infer the mechanism underlying a pattern of epistasis.

### Pairs of orthogonal perturbations to gene expression act independently upon enzyme levels

**Figure 3:**
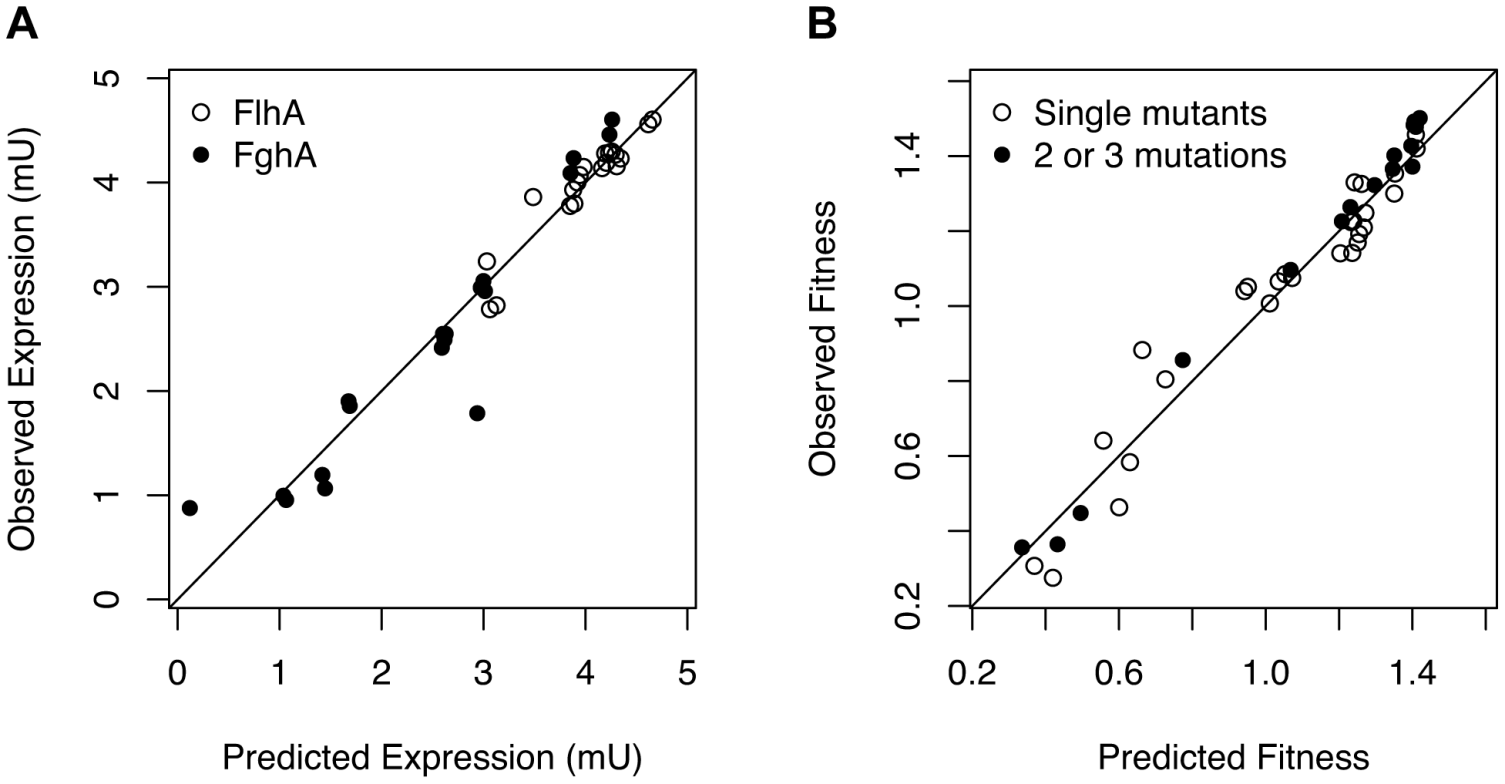
Mutations exert independent effects upon enzyme expression but a mechanistic model of benefits and costs of enzymes is required to predict combined effects on fitness. A) Expression levels (in mU) are well predicted by independent, multiplicative effects of each mutation on the FlhA and FghA enzymes. B) Using a kinetic model of catalysis and costs parameterized with data from the single mutants and inducible promoter constructs predicts the mutational combinations very well (R^2^ = 0.98).

What physiological factors underlie the strong antagonism observed between mutations affecting expression of the foreign GSH pathway? A first possibility is that mutational combinations lead to smaller changes in protein expression than expected from the single mutants and that such antagonistic behavior at the level of expression phenotypes merely propagated through as observed antagonism at the level of fitness. Because we used combinations that largely derived from pairing mutations that reduced expression per copy (Class A) with those that decreased plasmid copy number (Class B), our null hypothesis was that these mechanisms should act independently to alter expression, such that the expression level of an A+B mutant pair would simply be the product of these two values. Consistent with this prediction, enzyme levels were well described by the null model of multiplicative independence between paired perturbations (Figure 3A, Table S1). A simple linear model of log-transformed changes in enzyme levels as a function of the presence of the single mutations with no interaction terms explains much of the variation for both FlhA and FghA (adjusted-R^2^ = 0.85 (FlhA) and 0.86 (FghA)). This predictability can be seen in the high correlation between observed expression phenotypes for paired perturbations and those expected based upon the single changes.

### A fitness landscape model that incorporates benefits and costs predicts the fitness of mutational combinations

Since mutational combinations did not introduce epistatic interactions at the level of gene expression, we built a model of the fitness landscape based upon enzyme levels in order to ask how its shape would contribute to antagonism. Building upon earlier work on single enzymes [26] (Supplementary Text S2), we generated a model of the fitness landscape that calculates fitness as flux through the pathway above a threshold, minus the sum of two costs 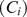:
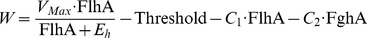
The hyperbolic expression for catalysis has been used before [40] to effectively describe the dependence of steady-state flux to the levels of a single enzyme and incorporates a “*V*_max_” term for the pathway, and an E*_h_* half-maximal enzyme level term. We only model FlhA concentration as beneficial to fitness even though FghA is absolutely required for growth on methanol [34]. This is because, over the parameter range of our perturbations, fitness appeared to rise monotonically with decreasing levels of FghA. This suggests FghA is a typical enzyme that has a low metabolic “control coefficient” [20], [21] and that it only limits catalysis at exceptionally low levels. None of our perturbations pushed FghA levels below 2%, and for comparison β-galactosidase levels in lactose-limited chemostats only impacted fitness significantly if they decreased activity to ≤1% [41].

The threshold flux term was added to the model to capture an unusual right-shift of the typical relationship between enzyme concentration and fitness observed with FlhA in these data, such that fitness approached zero even in the presence of measurable concentrations of functioning enzyme. We have observed similar behavior when manipulating levels of the analogous enzyme in the endogenous, tetrahydromethanopterin-dependent pathway for formaldehyde oxidation in WT (SM Carroll, CJM, unpublished). As both of these enzymes occur directly downstream of formaldehyde production, this threshold phenomenon may be explained by toxic effects of elevated steady-state formaldehyde concentrations at low enzyme levels. Finally, there are two cost terms for FlhA and FghA. The cost per molecule for each enzyme was treated as a linear function, consistent with prior work [26], [42].

**Figure 4:**
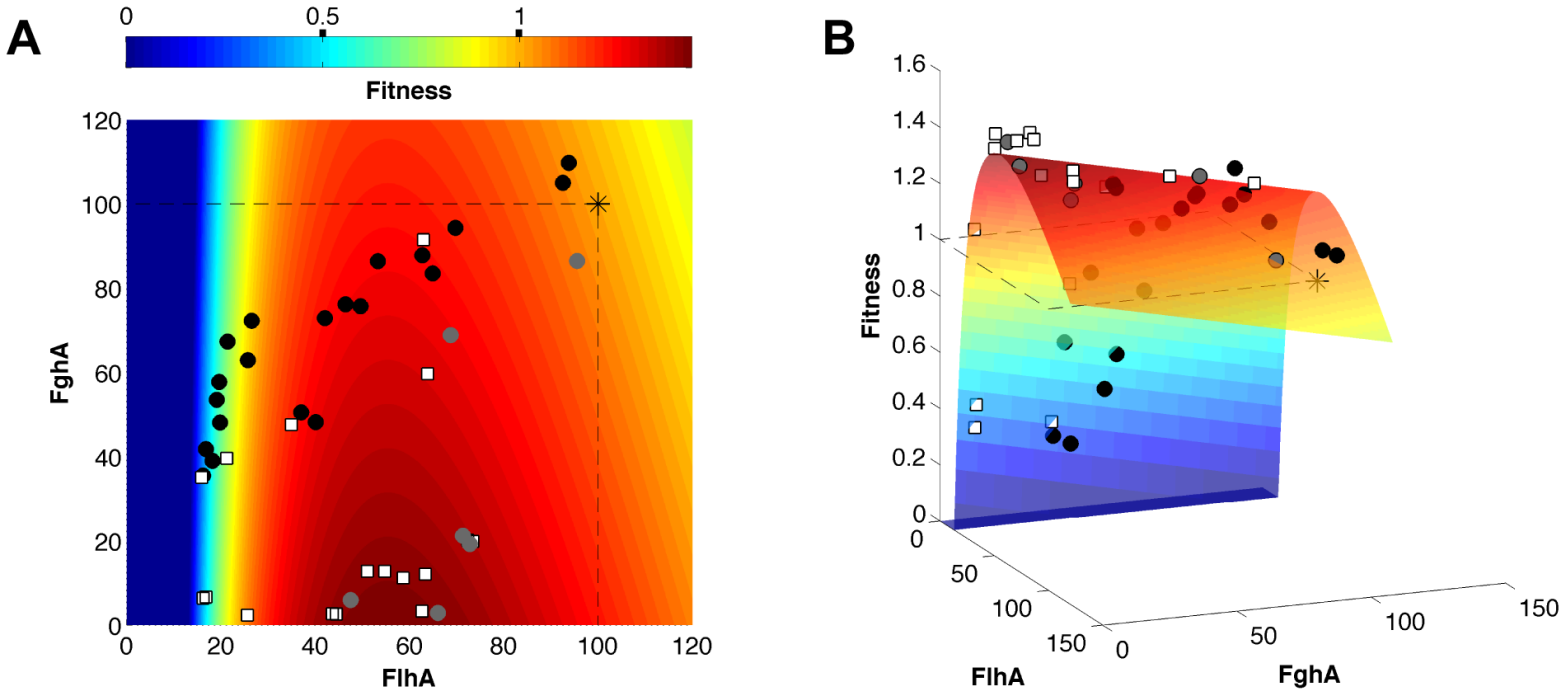
Fitness landscape of the GSH-linked pathway. A) A two-dimensional heatmap and B) three-dimensional surface showing the shape of the fitness landscape predicted by the model. The x and y axes are FlhA and FghA levels relative to ancestor (set at 100). Though the plasmid cost is included in the model, this fourth dimension was corrected for to allow a three-dimensional visualization (see Methods). Experimental data points indicate the ancestor (asterisk), single mutants (grey circles), mutational combinations (white squares), and inducible expression vectors (black circles).

The six parameters of this benefit - costs model were fit using the data from the EM ancestor, single mutants, as well as strains with inducible promoter plasmids (27 data points; Table S3). The inducible promoter plasmids contained a cumate-responsive repressor to modulate the levels of *flhA-fghA* from ancestral levels to lower values (Table S1). These data were critical for capturing the steep decline of the fitness landscape at low values of FlhA. The resulting benefit - costs model captured the curvature of the fitness landscape (Figure 4) and, unlike the simple multiplicative model, it was able to predict the 17 combinations of mutations that were not used for model fitting with high precision (R^2^ = 0.98) (Figure 3B). From the perspective of the ancestral genotype, in the model fitness rises gently with decreased expression of either enzyme, but then declines rapidly upon reaching catalytically-limiting levels of FlhA. A similar cliff exists for low values of FghA [34], but at enzyme levels beyond the range of our dataset and below the detection threshold of our enzyme assay method (see Methods).

### Beneficial mutations moved directly toward the global expression optimum rather than in the locally steepest direction on the fitness landscape

**Figure 5:**
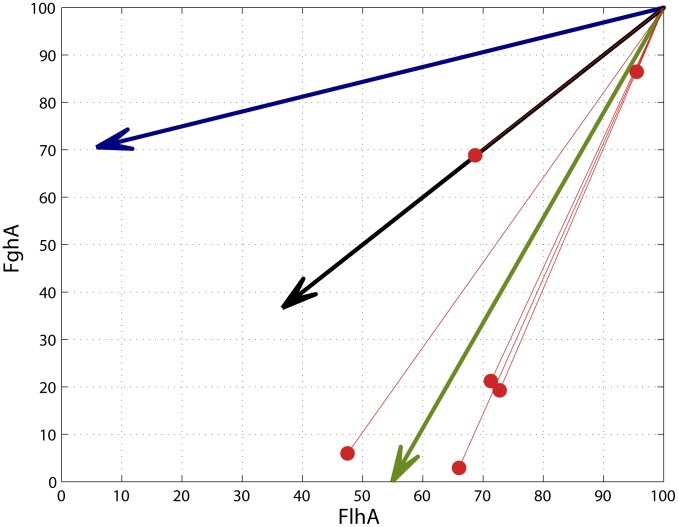
Adaptive mutations tended to move phenotypes toward the global optimum. The direction of phenotypic movements for strains with single mutations (red lines; enzyme levels of ancestor set to 100) are compared to the vectors indicating of the locally steepest fitness gradient for the ancestor (blue), complete 1∶1 correlation between phenotypes (black), and the global optimum (green). The direction of phenotypic movement for all single mutations was closer to the vector for the global optimum relative to the local gradient around the ancestor.

Our fitness landscape model that precisely maps phenotypes to fitness allowed us to explore how much local topography may have influenced the direction of phenotypic change during evolution by *de novo* mutations (Figure 5). We compared the changes in enzyme expression caused by single beneficial mutations relative to three factors: 1) the local gradient in the fitness landscape for the ancestor (greater decreases of FlhA versus FghA because the former is more costly), 2) the direct vector pointing to the global fitness optimum and 3) equal proportional changes between the enzymes which might be expected due to the physical constraint of their being expression from a single transcript. All mutations moved toward the global optimum rather than ascend in the phenotypically steepest direction on the local fitness landscape. Mutations B2 and B5 affected copy number but, through mechanisms we do not currently understand, led to greater decreases in FghA than FlhA. In contrast, B3 was directly along the line of equivalent change in both enzymes. This mutation was identified along with B2 as a plasmid haplotype, and this B2–B3 combination allowed this lineage to accomplish a similar phenotypic (and fitness) change as the other mutants.

## Discussion

We found that combining adaptive mutations that optimize expression of a two-enzyme pathway exhibited strong antagonistic interactions and sign epistasis. Fitness values of mutational combinations were generally less than expected relative to a null model of independent, multiplicative effects upon fitness. We further observed a negative relationship between *s* and ε for individual mutations. Other datasets of intragenic epistasis revealed similar trends between *s* and ε; however, this overall trend of epistasis (e.g., antagonism) does not imply a specific connection with properties of the individual mutations, such as *s*. For example, this trend will also arise as a consequence of regression to the mean when the beneficial mutations assayed are conditioned to be beneficial in the ancestral background and the effect of a mutation has a component that is independently distributed on each possible genetic background. Therefore, to extract biological insight from the quantitative relationship between *s* and ε , we must interrogate the mechanisms that lead to antagonistic epistasis.

The first possible explanation for antagonism in our data would be non-linearities in the way mutations combined to affect enzyme expression. However, as expected from having chosen combinations that combined class A mutations with those from class B, these orthogonal mechanistic effects resulted in independent effects on enzyme expression that were jointly well predicted with a simple, multiplicative model. As in this system, many ecologically relevant genes are encoded on plasmids whose regulation and gene dosage may both be effected by separate sets of mutations. More broadly, mutations that influence different traits that make joint contribution to a higher phenotype such as fitness are common. At the level of individual genes, for example, catalytic improvement of an enzyme often results from the joint contribution of mutations that improve protein stability and those that enhance kinetic parameters [43]–[45].

The second factor that could generate antagonism is the curvature of the underlying fitness landscape for gene expression. Recent theory has shown that almost any formulation of fitness based upon multiple underlying phenotypes will generate epistasis at the level of fitness, even when the mutations – as we observed here – do not interact epistatically on the underlying trait phenotypes [46]. Previous models have formulated fitness as a function of gene expression correctly predicted the evolution of optimal levels of gene expression [26]. Here we extended this model framework to multiple enzymes and used it for the first time to interpret beneficial mutational effects from phenotype to fitness, which we characterized both individually and in combination. Our fitness landscape model was able to predict the fitness values of the mutation combinations with high precision (R^2^ = 0.98). The asymmetry in the curvature of this fitness landscape results from the relatively gentle effects of expression costs relative to the sharp transition in fitness effects due to rate limitation upon catalysis [41]. This observed selection to maintain an intermediate optimum of enzyme levels is distinct from the selective neutrality on a catalytic plateau that was predicted by classical MCA analyses that did not incorporate expression costs [40].

**Figure 6:**
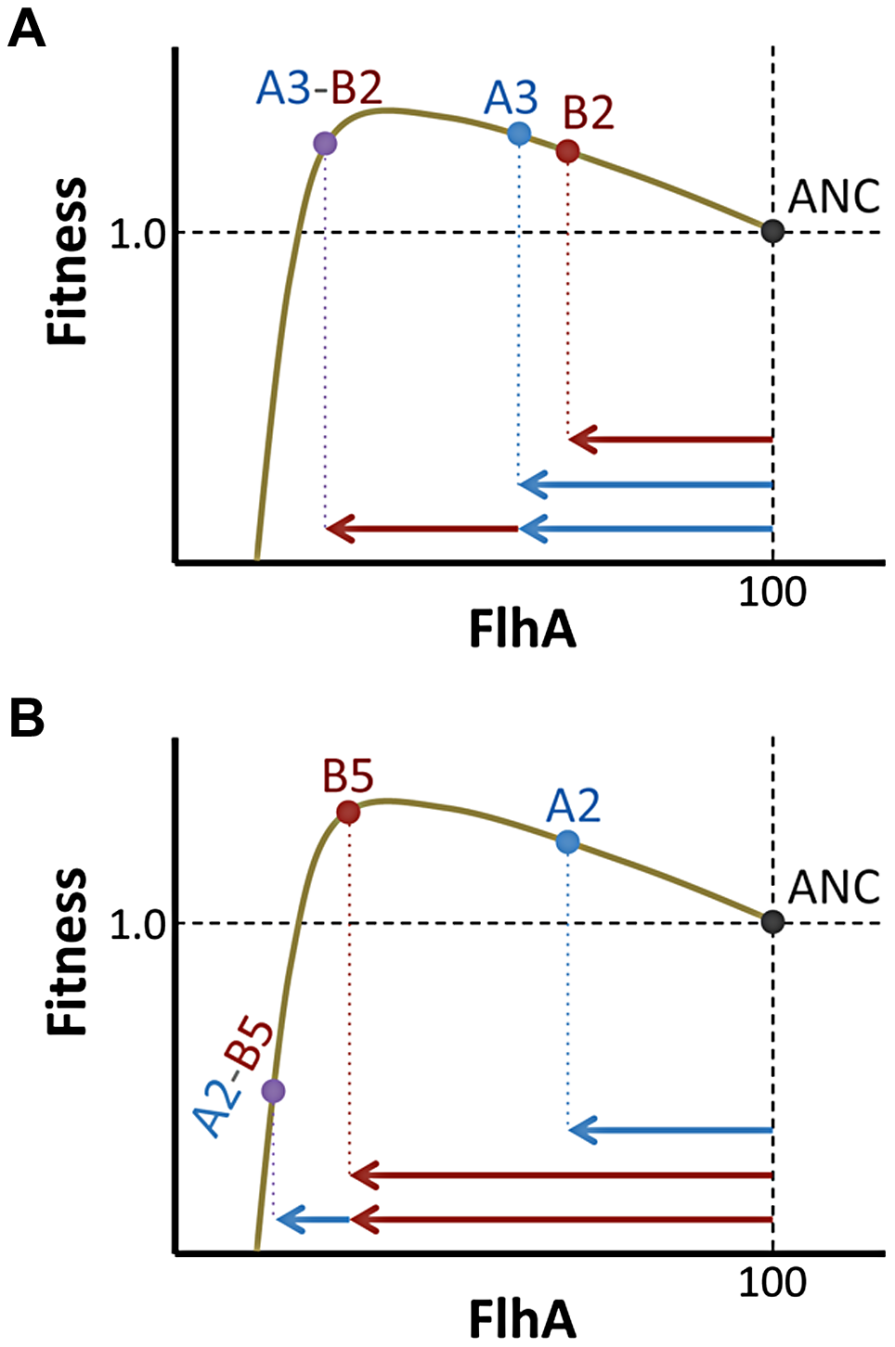
Phenotype and not just fitness value determines epistatic interactions. Mutations (A) A3 and (B) B5 had similar fitness values, but resided on opposite flanks of the optimal phenotype (shown here for simplicity as FlhA enzyme levels). In interactions with secondary beneficial mutations (B2 and A2, respectively), B5 fares worse due to being closer to the fitness cliff that occurs when FlhA catalysis becomes limiting.

Knowledge of the underlying fitness landscape allows us to understand aspects of the epistatic interactions not evident from fitness values alone. For example, we observed that the A3 and B5 mutations had fairly comparable individual fitness values (1.420±0.032 vs. 1.457±0.033; mean and 95% CI), but the former had three-fold more antagonistic epistasis than the latter (average = −0.46 vs. −1.34; t-test, p = 0.025; Figure 6). The modeled fitness landscape illuminates the underlying reason for this difference. Both mutations rest near the peak value of enzyme expression, but on opposite sides (B5 has 70% the level of FghA as A3). This poises B5 such that it is much more sensitive to further reductions in expression than A3. Thus, although these two mutations are essentially equivalent if one only considers their fitness values, their locations in phenotypic space change their likelihood for antagonism and sign epistasis.

One consequence of sign epistasis between mutations affecting the phenotypes like gene expression is a reduction of the benefit caused by recombination bringing beneficial mutations together into the same genome (i.e., Fisher-Muller model). This tradeoff between benefits and costs is inherent to gene expression, and thus results in stabilizing selection. These patterns of epistasis are likely very common, given the apparent ubiquity of stabilizing selection upon gene expression from microbes to primates [47]–[50].

With a complete fitness landscape defined by biochemical phenotypes, we can now interpret the genetic landscape in terms of what was accessible to individual mutations. In contrast to how selection acts upon standing genetic variation, the *de novo* mutations fixed in experimental populations ignored the phenotypically-local best direction of change and “jumped” towards the global optimum. This highlights that the classic quantitative genetics intuition of climbing in the direction of the steepest selection gradient [51], which is appropriate for small populations containing standing genetic variation, fails to capture the phenotypic potential of a sizeable pool of de novo mutations arising from large populations. In our case, it was not the large magnitude of expression change that was surprising, per se, but change in the ratio of their expression. Given that *flhA* and *fghA* are encoded on the same transcript, it was notable that all but one of the single mutations down-regulated FghA to a large extent while only cutting FlhA levels in half. Whereas this system allowed individual mutations to reach near-optimality, a multi-step trajectory was required for the directed evolution of the LacI repressor to reverse its regulatory logic [52]. In that system, the first round mutation simply broke the old logic to become constitutive, which in combination with two latter mutations allowed the “anti-LacI” phenotype to emerge and locate the fitness peak they predicted from a computational model. Our results suggest that relatively large moves in multi-phenotype space can emerge as winners, provided the genetic architecture at least allows rare mutations to achieve this possibility.

Finally, the near optimal expression levels of these beneficial mutations becomes even more remarkable when considering that this optimization did not happen in isolation, but in adapting populations that contained many beneficial mutations simultaneously [33], [34], [53]. In varying environments, such diversity in large microbial populations may lead to genetically complex adaptation such as stable polymorphisms [54]. In a stable environment, this diversity leads to ‘clonal interference’ [55], a type of serial fixation that effectively sorts for mutations of the greatest effect amongst what was possible. This would have impacted the mutations that affected GSH pathway expression in two ways. Firstly, there were many different genetic solutions to reducing expression of these enzymes [34]. One type in particular - the Class C mutations that resulted from integration of the introduced plasmid into the host chromosome – occur at very high rates and emerged to detectable levels repeatedly, up to 17 times per population [33]. These mutations confer ∼⅔ the benefit of the Class A and B mutations [34], however, and were only found to rise to fixation in three of eight populations despite more than 100 observed occurrences [33]. In this regard, clonal interference aids finding optimal solutions by allowing only the best individual mutations to fix. Recently, however, it has been shown that fixation probability of contending mutations is only partly dependent upon their individual effect because they commonly hitchhike with other beneficial mutations present [56]–[58]. This leads to a second effect of clonal interference, which is competition between lineages carrying beneficial mutations affecting distinct phenotypic processes. Indeed, the ancestral genotype faced a variety of phenotypic challenges besides just optimizing expression of the GSH pathway [14], [59], [60]. Some of these mutations in other loci had beneficial effects up to 3× larger than those described here [10] and were segregating at the same time as mutations affecting expression of the GSH pathway [33], [53]. Even with so much turmoil in the populations, the eventual winners discovered nearly optimal solutions to this local, two-enzyme expression optimization in order to win the battle for fixation. Population size thus contributed to the fixation of optimal solutions by both increasing the number of mutations occurring and escaping drift in the first place, and by facilitating competition between multiple potential solutions. These factors conspired to allow selection to reward – when mutationally possible – lineages that made long-range, lucky jumps to distant peaks on the phenotypic landscape.

## Materials and Methods

### Experimental evolution and growth conditions

The EM strain was generated previously by deleting the *mptG* gene of *M. extorquens* AM1 in the white strain WT CM502 [61] lacking carotenoid pigments due to an unmarked mutation in *crtI* (encoding phytoene desaturase) [62], followed by introduction of pCM410 [10]. Eight replicate populations seeded by the EM strain were grown in 9.6 ml methanol (15 mM) minimal media incubated in a 30°C shaking incubator at 225 rpm. Populations were transferred to fresh media at a 1/64 dilution rate (thus six generations per growth cycle, N_final_≈10^9^) and propagated for 600 generations. One liter of minimal media consists of 100 ml of phosphate buffer (25.3 g of K_2_HPO_4_ and 22.5 g of NaH_2_PO_4_ in 1 liter of deionized water), 100 ml of sulfate solution (5 g of (NH_4_)_2_SO_4_ and 0.98 g of MgSO_4_ in 1 liter of deionized water), 799 ml of deionized water, and 1 ml of trace metal solution. One liter of the trace metal solution consists of 100 ml of 179.5 mM FeSO_4_, 800 ml of premixed metal mix (12.738 g of EDTA disodium salt dihydrate, 4.4 g of ZnSO_4_·7H_2_O, 1.466 g of CaCl_2_·2H_2_O, 1.012 g of MnCl_2_·4H_2_O, 0.22 g of (NH_4_)_6_Mo_7_O_24_·4H_2_O, 0.314 g of CuSO_4_·5H_2_O, and 0.322 g of CoCl_2_·6H_2_O in 1 liter of deionized water, pH 5), and 100 ml of deionized water [14].

### Plasmid and strain construction

All strains and plasmids used are indicated in Table S2. All plasmids constructed in this study were maintained in *E. coli* 10-beta strain (New England Biolabs) and were transferred to *M. extorquens* via electroporation [63] or tri-parental mating with the helper strain pRK2073 [64]. Plasmid DNA in *E. coli* was extracted using the QIAprep Spin MiniPrep Kit (Qiagen). The *P_mxaF_* expression vector pCM160 [65], its variant pCM410 in the EM strain expressing the *flhA*-*fghA* cassette [10], and the cumate-inducible vector pHC112 expressing the *flhA-fghA* cassette [34] have been described previously. In order to combine Class A and B mutations which accumulated on separate pCM410 derivatives during experimental evolution of the EM strain (Figure 1B, Table S1), Class B mutations (from pCM410^B2B3^, pCM410^B2^, pCM410^B3^, and pCM410^B5^) were moved to pCM410 derivatives bearing Class A mutations (pCM410^A1^, pCM410^A2^, and pCM410^A3^) through the procedures delineated below. Fragments containing B2–B3, B2, and B3 mutations were first obtained by digesting pCM410^B2B3^, pCM410^B2^, or pCM410^B3^ with *Sfi*I and *Nhe*I. These were then ligated into the plasmid backbone of pCM410^A1^, pCM410^A2^, or pCM410^A3^ cut with the same enzymes. A fragment containing the B5 mutation was obtained by digesting of pCM410^B5^ with *Sfi*I and *Sex*AI and then ligated into the plasmid backbone pCM410^A1^, pCM410^A2^, or pCM410^A3^ cut by the same enzymes. The above procedures were also applied to introduce B2–B3, B2, B3, and B5 mutations into pHC112 in order to generate pHC112 derivatives that vary in their plasmid copy number.

### Fitness assays

Fitness assays were performed by a previously described procedure [34]. Strains were first physiologically acclimated through one 4-day growth cycle in 9.6 ml of minimal media supplemented with 15 mM methanol. In addition, for strains bearing cumate-inducible promoter plasmids (pHC112 derivatives), different concentrations of cumate (Table S1) were added to growth media to modulate the expression of FlhA and FghA enzymes. After this acclimation phase, each of these strains was mixed with a fluorescent variant (CM1232) of the EM ancestor [10] by a 1∶1 volume ratio, diluted 1/64 into 9.6 ml of fresh growth media, and incubated in a 30°C shaking incubator at 225 rpm. The ratios of the two populations before (R_0_) and after (R_1_) competitive growth were quantified by a LSR II flow cytometer (BD Biosciences) for at least 50000 cell counts per sample. The forward scatter threshold of LSRII was adjusted to 300 to ensure unbiased detection of the test and reference strains despite their potential differences in cell size. Fitness values (W) relative to the reference strain were calculated by a previously described equation assuming an average of 64-fold size expansion of mixed populations during competitive growth [35]:
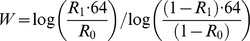
In order to convert to absolute differences in growth rate, the EM ancestor grows under these conditions with a growth rate of 0.0654+/−0.0016 h^−1^ [10].

### Enzyme assays

The activities of FlhA [66] and FghA [67] were assayed in three replicates as described using cells harvested from mid-exponential phase cultures. Cells were collected through centrifugation at 10,000× *g* for 10 min, frozen at −80°C, and used for enzyme assays within a week. Right before assays frozen cell pellets were suspended in 50 mM Tris-HCl buffer (pH 7.5) and physically disrupted in tubes containing Lysing Matrix B and shaken at speed 6.0 m/s on a FastPrep®-24 bead beater (MP Biomedicals) for 40 seconds. Insoluble debris in the cell lysate was removed by centrifugation at 13,000× *g*, 4°C for 15 min. The total protein concentration of the cell lysate was quantified using the Bradford method [68]. Kinetic analysis of FlhA and FghA activities over 10 min at 30°C was performed in 200 µl reaction mixtures using a SpectraMax M5 Plate Reader (Molecular Devices).

### Quantification of plasmid copy numbers

The copy number of pCM410 derivatives in *M. extorquens* was quantified by a real-time PCR approach described previously [34]. Briefly genomic DNA of *M. extorquens* from mid-exponential phase cultures was extracted by an alkaline lysis method [69]. Detection of plasmid DNA was targeted at the *kan* gene using primers HC410p18 (5′-GAAAACTCACCGAGGCAGTTCCATAG-3′) and HC410p19 (5′-TCAGTCGTCACTCATGGTGATTTCTCA-3′). Detection of chromosomal DNA was targeted at the *rpsB* gene (encoding the 30S ribosomal protein S2) in the chromosome META1 using primers HCAM111 (5′-TGACCAACTGGAAGACCATCTCC-3′) and HCAM113 (5′-TTGGTGTCGATCACGAACAGCAG-3′). Real-time PCR experiments were performed in three replicates with the PerfeCTa SYBR Green SuperMix (Quanta Biosciences) on a DNA Engine Opticon2 (MJ Research), and the average threshold cycle (Ct) of each PCR reaction was determined using the Opticon Monitor v. 2.02 software (MJ Research). Each real-time PCR reaction contained 25 ng of genomic DNA extracted from various strains and *kan*- or *rpsB*-specific primers. To establish a standard curve (SC) of plasmid copy numbers, 1, 0.1, 0.01, and 0.001 ng of pCM410 (equivalent to 9.09×10^7^, 9.09×10^6^, 9.09×10^5^, and 9.09×10^4^ plasmid molecules, respectively) were mixed with 25 ng of genomic DNA (equivalent to 3.03×10^6^ genome copies) of the plasmid-less, white WT *M. extorquens* (CM502) [61]. The standard curve is a plot of ΔCt (i.e. Ct*_kan_*–Ct*_rpsB_*) versus plasmid molecules on a log_2_ scale. For each strain, by interpolating its ΔCt value against the SC the absolute quantity of plasmid DNA can be estimated using the following equation:
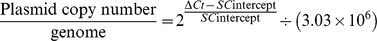



### Model fitting and comparison

Multiplicative models predicting fitness or gene expression were fit and assessed as standard linear models following a log transformation of the response variable. The model for the fitness landscape was fit using a non-linear routine in Matlab. The raw data as well as commented code in Matlab and R that completely recreates the analysis and figures has been deposited at www.datadryad.org (doi:10.5061/dryad.8hb23).

## Supporting Information

Figure S1 Trends of epistasis as a function of selective coefficient. The epistasis values on fitness were calculated for every mutational combination a mutation with a given value of *s* was found in. The datasets represent: intragenic epistasis during single adaptive trajectories of (A) *M. extorquens* [10] or (B) *E. coli* [11], (C) intragenic and intergenic combinations of independent mutations from the bacteriophage ID11 [12], or (D,E) two datasets of intragenic combinations of *E. coli* β-lactamase alleles [17], [38]. Note that ID11 data were given as doublings per hour, and we therefore exponentiated with base two. These transformed data, as well as the other four data sets, were treated as multiplicative fitnesses; these values were normalized by dividing by the ancestor fitness, and the epistasis of mutation *i* in background *g* was calculated as ε*_ig_* = *W_ig_*−*W_i_*×*W_g_*.
(DOCX)

Text S1 Analysis of bias in epistasis as a function of selection coefficient.
(DOCX)

Text S2 Derivation of the fitness model.
(DOCX)

Table S1 Genotypes and phenotypes of pCM410, pHC112, and their derivatives.
(DOCX)

Table S2 Bacterial strains and plasmids.
(DOCX)

Table S3 The value of fitted parameters in the fitness landscape model.
(DOCX)
