# Supplementary material for: Mapping the Fitness Landscape of Gene Expression Uncovers the Cause of Antagonism and Sign Epistasis between Adaptive Mutations
Source: PLoS Genet. 2014 Feb 27;10(2):e1004149. doi: 10.1371/journal.pgen.1004149 (PMC3937219; doi:10.1371/journal.pgen.1004149)
Supplement: Figure S1 — Trends of epistasis as a function of selective coefficient. The epistasis values on fitness were calculated for every mutational combination a mutation with a given value of s was found in. The datasets represent: intragenic epistasis during single adaptive trajectories of (A) M. extorquens [10] or (B) E. coli [11], (C) intragenic and intergenic combinations of independent mutations from the bacteriophage ID11 [12], or (D,E) two datasets of intragenic combinations of E. coli β-lactamase alleles [17], [38]. Note that ID11 data were given as doublings per hour, and we therefore exponentiated with base two. These transformed data, as well as the other four data sets, were treated as multiplicative fitnesses; these values were normalized by dividing by the ancestor fitness, and the epistasis of mutation i in background g was calculated as εig = Wig−Wi×Wg. (DOCX) [file pgen.1004149.s001.docx]

**Figure S1. Trends of epistasis as a function of selective coefficient.**
