# Supplementary material for: Mapping the Fitness Landscape of Gene Expression Uncovers the Cause of Antagonism and Sign Epistasis between Adaptive Mutations
Source: PLoS Genet. 2014 Feb 27;10(2):e1004149. doi: 10.1371/journal.pgen.1004149 (PMC3937219; doi:10.1371/journal.pgen.1004149)
Supplement: Text S1 — Analysis of bias in epistasis as a function of selection coefficient. (DOCX) [file pgen.1004149.s002.docx]

**Text S1. Analysis of bias in epistasis as a function of selection coefficient.**

The slope of the relationship between the selective coefficient of a mutation in the ancestral background, and its epistatic interactions with other beneficial mutations, seems intuitively sufficient to reveal a trend toward increasing synergistic or antagonistic epistasis. If mutations with larger beneficial effects in the ancestor combine more poorly with other mutations, this slope will be negative; such negative relationships appear consistent with an explanation of diminishing returns, where the benefit of a mutation decreases monotonically with the fitness of the genetic background in which it is assayed. However, recent work in a computational model of fitness landscapes has suggested that ascertainment bias in evolution experiments can produce statistical artifacts that may be hard to distinguish from the patterns expected with diminishing returns [39]. Here we explore a simple, illustrative model to show the fitness landscapes without diminishing returns can show spurious negative correlations between the benefit of mutations in the ancestor and their fitness effects in combination.

Our simplified fitness landscape model starts with *L* sites, each of which permits a single alternative allele. By assumption, each of these mutations has an inherent, or mean effect, which is independent of the genetic background, and a random, or epistatic, effect, which reflects the influence of the genetic background on that allele’s fitness contribution. The inherent effect of a locus *i*, μ_i_, is drawn independently and identically from a Gaussian distribution with mean zero and standard deviation m_sd_. Random effects have a mean of zero and a standard deviation which is specific for each locus; these standard deviations, σ_i_, are drawn independently and identically from an exponential with mean r_sd_. The random effects for loci *i* in background g are then drawn from a Gaussian with mean zero and standard deviation σ_i_. Let N(a, b) stand for a Gaussian draw with mean a and standard deviation b and E (c) for an exponential draw with mean c; then, the inherent and random effects of the mutation at locus *i* are:

μ_i_ = N(0, m_sd_) Eq. 1

σ_i_ = E(r_sd_) Eq. 2

If the ancestor has a fitness of one, then the fitness of the genotype with a mutation at locus *i* is:

w_i_ = 1 + μ_i_ + N(0, σ_i_) = 1 + μ_i_ + r_i_ Eq. 3

where r_i_ is the random effect in the ancestor. Assuming that fitness is calculated on a multiplicative scale, the fitness of the genotype with mutations at both loci *i* and *j* is:

w_ij_ = (1 + μ_i_ + r_ij_)(1 + μ_j_ + r_ji_) Eq. 4

where r_ij_ is the random effect of mutation *i* in the background containing *j*, and r_ji_ is the random effect of mutation *j* with *i*. We can then calculate epistasis between mutations at loci *i* and *j* by subtracting the expected fitness from the of the double mutant from its actual fitness, as calculated with Eq. 4.

ε_ij_ = w_ij_ - w_i_w_j_ Eq. 5

Epistasis, as represented in this model, is not biased toward antagonism at higher fitnesses; we therefore do not expect to see signatures of diminishing returns from experiments conducted on these model landscapes. If a trend toward negative regressions of ε on *s* of mutations is observed for beneficial mutations in this model, then we can conclude that the process of selection makes these regressions vulnerable to spurious indications of diminishing returns.

To generate each set of beneficial mutations, we first assign fitness effects in the ancestor using Eq. 3. Then, we select a group of *n* beneficial mutations with a simplified model of an evolutionary process. This selection step reflects an ascertainment bias: mutations that escape genetic drift and reach fixation are likely to be very beneficial on the background of the ancestor, and are therefore likely to exhibit a regression to the mean when tested on other genetic backgrounds. To illustrate the effects of this potential bias, we consider two procedures for selecting beneficial mutations. In the first, which we will call *weak selection*, all beneficial mutations are equally likely to be chosen. In the second, labeled *strong selection*, beneficial mutations are chosen proportionally to their selective coefficients.

In each simulated experiment, six unique beneficial mutations are chosen according to either the weak or strong selection procedure, and the slope of the relationship between *s* and ε is determined by linear regression. These experiments are replicated 10,000 times for each parameter values, and we report the mean slope and the fraction of slopes that are negative. Because of the nature of selection in our model, only the relative magnitudes of selective coefficients are important; we therefore only consider the ratio m_sd_:r_sd_.

Mean regression slopes, and the fraction of slopes that are negative, for strong selection simulations. Dashed lines indicate the unbiased expectations. Parameter values are m_sd_ = 0.01, r_sd_ = 0.05 for the ratio 0.2, m_sd_ = 0.05, r_sd_ = 0.05 for the ratio 1, and m_sd_ = 0.05, r_sd_ = 0.01 for the ratio 5.

The above figure summarizes the bias toward negative slopes between selective coefficients in the ancestor and epistasis in pairs for strong selection. For all parameter values, regression slopes are more likely to be negative. This reflects the sorting effects of selection, where fixed mutations are likely to be unusually good on the background in which they were selected, relative to other genetic backgrounds. This bias is more severe when the number of loci is large and the magnitude of random effects is large compared to mean effects. The results for the weak selection model are similar with two differences: biases are slightly smaller, and the effect of *L* is negligible (data not shown). These complex interactions between parameters, which depend on aspects of both the fitness landscape itself and the population process of selecting the mutations, highlight the difficulty in attempting to correct for these biases.
