# Supplementary material for: Mapping the Fitness Landscape of Gene Expression Uncovers the Cause of Antagonism and Sign Epistasis between Adaptive Mutations
Source: PLoS Genet. 2014 Feb 27;10(2):e1004149. doi: 10.1371/journal.pgen.1004149 (PMC3937219; doi:10.1371/journal.pgen.1004149)
Supplement: Text S2 — Derivation of the fitness model. (DOCX) [file pgen.1004149.s003.docx]

**Supplementary Text S2. Derivation of the fitness model.**

**Comparison of current benefit – cost model to previous analysis in *M. extorquens*.**

The present formulation of fitness as a function of benefits and costs bears some similarities to a prior phenomenological model for *M. extorquens* [10], but there are substantial differences. In order to analyze epistasis amongst four beneficial mutations that occurred in a single evolving lineage of the EM strain, we had noted that enzyme expression cost was a substantial component of adaptation and built a simple model of fitness around considering the impact cost would have. Three of the four alleles identified in an evolved (EVO) strain appeared to increase fitness at least partly by reducing this cost. Therefore, like models for a single enzyme [26], we partitioned fitness into a ‘benefit’ component *b_0_*, analogous to a single conglomerate ‘enzyme activity’ that sets the rate of energy extracted from the substrate to generate biomass; and a cost *c_0_*, encompassing a fixed amount of energy diverted to deal with over-expression of the foreign pathway. Thus the fitness of the ancestral strain can be written, as *W_0_* = *b_0_-c_0_* = 1. We hypothesized that new alleles could modify benefit and cost by multiplicative factors (*λ_i_* and *θ_i_*, respectively), giving rise to a fitness *W_i_* = *λ_i_b_0_-θ_i_c_0_*. A successive allele *j*, on top of the background of mutant *i*, is similarly assumed to act multiplicatively on the benefit and cost components, yielding a fitness *W_ij_* = *λ_i_λ_j_b_0_-θ_i_θ_j_c_0_*.

There are three primary differences between the model described above and the one developed in the current work. First, we measured changes in the enzyme levels themselves and using these as an intermediate level phenotype that is the basis of calculating fitness. This contrasts with the above model that only considered the fitness values of various strains and abnormal cellular morphology as a proxy for costs. Second, we distinguish between the two enzymes rather than lumping them together, for breaking the original correlation in their expression was critical for adaptation [34]. Third, we explicitly describe the pleiotropy that links the consistent, predictable changes in benefit *and* costs that occur when expression of FlhA is altered. The above model contains the potential for pleiotropy in that each mutation could have values of *λ_i_* and *θ_i_* that both differ from 1, but here that shape is established based upon a simplification of MCA [40]. It is because of these linkages to the underlying physiology that the current model can generate accurate predictions of fitness for mutational combinations not used in the training set (R^2^ = 0.98).

**Mechanistic derivation of the fitness model from Metabolic Control Analysis**

Our fitness model is a biologically inspired but ultimately empirical description of the effect of the measured enzyme levels on fitness. However, it is also possible to directly derive this model from an underlying mechanistic model, as explained in this supplemental text. We would like to gratefully acknowledge that this derivation was originally provided by an anonymous reviewer of this manuscript.

Consider a model where fitness ($W$) was proportional to the flux through a pathway, $(J)$, with linear penalties for the concentration of a toxic metabolic intermediate ($S_{i}$) as well as the concentration of the enzymes $FlhA$ and $FghA$ along this pathway, such that:

$$W=\beta_{0}J-\beta_{1}S_{i}-\beta_{2}FlhA-\beta_{3}FghA$$

In our conception of this model, the toxic metabolite is formaldehyde, which appears upstream of the enzymatic reactions catalyzed by *FlhA* and *FghA* in the pathway of methanol metabolism in this organism.

Additionally, imagine that the pathway that determines fitness is a linear pathway that contains the toxic intermediate $S_{i}$ as an intermediate step. This pathway begins at a substrate $S_{1}$ whose concentration is fixed, and ends at the irreversible reaction catalyzed by $E_{n+1}$. Further, assume that this pathway is governed by mass action kinetics and that the kinetic rates of the forward and reverse reactions do not change during the evolution experiment.


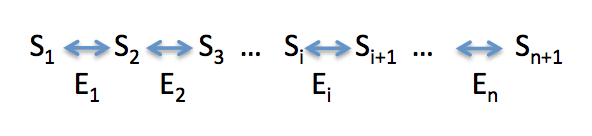


One can show that this model can be reduced to our model for fitness. This derivation depends on many classic results from metabolic control theory, and if these are unfamiliar to the reader they are encouraged to consult a reference book before examining this result (or Appendix C in [20]).

First make the following definitions:

$$k_{x}=Forward rate through the xth reaction$$

$$k_{-x}=Reverse rate through the xth reaction$$

$$q_{x}=\frac{k_{x}}{k_{-x}}=ratio of rates$$

$$K_{j,k}=\prod_{x=j}^{k} q_{x}$$

$$e_{x}=Concentration of enzyme X$$

$$E_{X}=e_{x}k_{x}=enzyme times forward rate constant$$

Then for a linear pathway of length $n$ reactions, the flux through the pathway will be equal to:

$$J=\frac{S_{1}}{\sum_{x=1}^{n} \frac{1}{K_{1,x}E_{x}}}$$

Because the flux through the entire pathway is equal to the flux from the toxic intermediate as well, we can set these two fluxes to be equal and solve for $S_{i}$.

$$J=\frac{S_{1}}{\sum_{x=1}^{n} \frac{1}{K_{1,x}E_{x}}}=\frac{S_{i}}{\sum_{x=i}^{n} \frac{1}{{K_{i,x}E}_{x}}}$$

$$S_{i}=\frac{S_{1}\sum_{x=i}^{n} \frac{1}{{K_{i,x}E}_{x}}}{\sum_{x=1}^{n} \frac{1}{K_{1,x}E_{x}}}$$

Substituting back in to our original equation for fitness we obtain:

$$W=\beta_{0}J-\beta_{1}S_{i}-\beta_{2}e_{FlhA}-\beta_{3}e_{FghA}$$

$$W=\beta_{0}\left( \frac{S_{1}}{\sum_{x=1}^{n} \frac{1}{K_{1,x}E_{x}}} \right)-\beta_{1}\left( \frac{S_{1}\sum_{x=i}^{n} \frac{1}{{K_{i,x}E}_{x}}}{\sum_{x=1}^{n} \frac{1}{K_{1,x}E_{x}}} \right)-\beta_{2}e_{FlhA}-\beta_{3}e_{FghA}$$

$$W=S_{1}\left( \frac{\beta_{0}-\beta_{1}\sum_{x=i}^{n} \frac{1}{{K_{i,x}E}_{x}}}{\sum_{x=1}^{n} \frac{1}{K_{1,x}E_{x}}} \right)-\beta_{2}e_{FlhA}-\beta_{3}e_{FghA}$$

Note that: $\frac{1}{K_{i,x}}=\frac{K_{1,i}}{K_{1,x}}$ and that the control coefficient for an enzyme in a pathway are equal to:

$$C_{x}=\frac{\left( \frac{e_{x}}{E_{x}K_{1,x}} \right)}{\left( \sum_{z=1}^{n} \frac{e_{x}}{E_{z}K_{1,z}} \right)}$$

As before $e_{x}$ is the concentration of enzyme for the $xth$step. By rearrangement one can then obtain:

$$W=S_{1}\left( \frac{\beta_{0}-\beta_{1}\sum_{x=i}^{n} \frac{K_{1,i}}{{E_{x}K}_{1,x}}}{\sum_{x=1}^{n} \frac{1}{K_{1,x}E_{x}}} \right)-\beta_{2}e_{FlhA}-\beta_{3}e_{FghA}$$

$$W=S_{1}\left( \frac{\beta_{0}}{\sum_{x=1}^{n} \frac{1}{K_{1,x}E_{x}}}-\beta_{1}K_{1,i}\left( \sum_{x=i}^{n} \left( \frac{\frac{1}{{K_{1,x}E}_{x}}}{\sum_{x=1}^{n} \frac{1}{K_{1,x}E_{x}}} \right) \right) \right)-\beta_{2}e_{FlhA}-\beta_{3}e_{FghA}$$

$$W=S_{1}\left( \frac{\beta_{0}}{\sum_{x=1}^{n} \frac{1}{K_{1,x}E_{x}}}-\beta_{1}K_{1,i}\sum_{x=i}^{n} \frac{C_{x}}{e_{x}} \right)-\beta_{2}e_{FlhA}-\beta_{3}e_{FghA}$$

We now scale all the enzyme concentrations except *FlhA* and *FghA* to be equal to 1 (as we are modeling their concentrations for different mutational combinations as effectively constant), and define two constants for notational ease that are related to the control coefficients as:

$$a=\frac{C_{FlhA}}{e_{FlhA}}+\frac{C_{FghA}}{e_{FghA}}+\sum_{x>i;x\neq flhA;x\neq fghA} C_{x}$$

$$b=\sum_{x\neq flhA;x\neq fghA} \frac{1}{K_{1,x} E_{x}}$$

Fitness can then be rearranged as:

$$W=S_{1}\left( \left( \frac{\beta_{0}}{b+\frac{1}{K_{1,FlhA} E_{FlhA}}+\frac{1}{K_{1,FghA} E_{FghA}}} \right)-\beta_{1}K_{1,i}\left( a+\frac{C_{FlhA}}{e_{FlhA}}+\frac{C_{FghA}}{e_{FghA}} \right) \right)-\beta_{2}e_{FlhA}-\beta_{3}e_{FghA}$$

Assume $c_{FghA}$ is much smaller than $c_{FlhA}$ (justifiable given the observation that this enzyme could be dropped almost to negligible concentrations relative to wild-type). This implies that: $\frac{1}{k_{FlhA}e_{FlhA}}\gg\frac{1}{k_{FghA}e_{FghA}}$, and justifies ignoring these *FghA* terms leading to:

$$W=S_{1}\left( \left( \frac{\beta_{0}}{b+\frac{1}{K_{1,FlhA} E_{FlhA}}} \right)-\beta_{1}K_{1,i}\left( a+\frac{C_{FlhA}}{e_{FlhA}} \right) \right)-\beta_{2}e_{FlhA}-\beta_{3}e_{FghA}$$

$$W=\left( \left( \frac{S_{1}\beta_{0}}{b+\frac{1}{K_{1,FlhA} E_{FlhA}}} \right)-\left( S_{1}\beta_{1}K_{1,i}a+\frac{S_{1}\beta_{1}K_{1,i}C_{FlhA}}{e_{FlhA}} \right) \right)-\beta_{2}e_{FlhA}-\beta_{3}e_{FghA}$$

$$W=\frac{S_{1}\beta_{0}}{b+\frac{1}{K_{1,FlhA} E_{FlhA}}}-S_{1}\beta_{1}K_{1,i}a-e_{FlhA}\left( \beta_{2}+\frac{S_{1}\beta_{1}K_{1,i}C_{FlhA}}{\left( e_{FlhA} \right)^{2}} \right)-\beta_{3}e_{FghA}$$

$$W=\frac{\frac{{e_{FlhA}S}_{1}\beta_{0}}{b}}{e_{FlhA}+\frac{1}{{k_{FlhA}K}_{1,FlhA}b}}-S_{1}\beta_{1}K_{1,i}a-e_{FlhA}\left( \beta_{2}+\frac{S_{1}\beta_{1}K_{1,i}C_{FlhA}}{\left( e_{FlhA} \right)^{2}} \right)-\beta_{3}e_{FghA}$$

Renaming the constants:

$$W=\frac{e_{FlhA}\gamma_{0}}{e_{FlhA}+\gamma_{1}}-\gamma_{3}-e_{FlhA}\left( \gamma_{4}+\frac{\gamma_{5}}{\left( e_{FlhA} \right)^{2}} \right)-\gamma_{6}e_{FghA}$$

And finally, as our model is designed to explore data where the enzyme concentration of *FlhA* is decreasing relative to the wildtype ($e_{FlhA}$ < 1.0) we can regard the term $\frac{\gamma_{6}}{\left( e_{FlhA} \right)^{2}}$ as relatively less important over the parameter range we are fitting (or that the penalty term in parenthesis on the right can be approximated by a simple linear penalty), leading to our model for fitness:

$$W=\frac{e_{FlhA}\gamma_{0}}{e_{FlhA}+\gamma_{1}}-\gamma_{3}-{\gamma_{4}e}_{FlhA}-\gamma_{6}e_{FghA}$$
