# Supplementary material for: Mapping the Fitness Landscape of Gene Expression Uncovers the Cause of Antagonism and Sign Epistasis between Adaptive Mutations
Source: PLoS Genet. 2014 Feb 27;10(2):e1004149. doi: 10.1371/journal.pgen.1004149 (PMC3937219; doi:10.1371/journal.pgen.1004149)
Supplement: Table S1 — Genotypes and phenotypes of pCM410, pHC112, and their derivatives. (DOCX) [file pgen.1004149.s004.docx]

**Table S1.** **Genotypes and phenotypes of pCM410, pHC112, and their derivatives.**

^a^The nucleotide positions of mutations on pCM410 (Genbank Accession no. FJ389188) are indicated in parenthesis.

^b^The concentrations of cumate (μM) applied to induce expression of the *flhA-fghA* cassette in pHC112 and its derivatives.

^c^Phenotypes during growth in methanol (15 mM) minimal media supplemented. Data are reported as means and 95% confidence intervals of three independent measurements. Plasmids were quantified as copies per genome. Enzyme activities of FlhA and FghA were measured as the amount of substrate being catalyzed per milligram of cellular protein per second (mM sec^-1^ mg^-1^).
