# Supplementary material for: Mapping the Fitness Landscape of Gene Expression Uncovers the Cause of Antagonism and Sign Epistasis between Adaptive Mutations
Source: PLoS Genet. 2014 Feb 27;10(2):e1004149. doi: 10.1371/journal.pgen.1004149 (PMC3937219; doi:10.1371/journal.pgen.1004149)
Supplement: Table S2 — Bacterial strains and plasmids. (DOCX) [file pgen.1004149.s005.docx]

**Table S2.** **Bacterial strains and plasmids.**

| Strain or plasmid | Description | Source or reference |
| --- | --- | --- |
| **Strains** |  |  |
| CM502 | Wild-type, *crtI*^502^ | [61] |
| CM624 | *crtI*^502^, Δ*mptG* | [10] |
| CM701 | Δ*mptG*, pCM410 | [10] |
| CM702 | *crtI*^502^, Δ*mptG*, pCM410 | [10] |
| CM1232 | *crtI*^502^*, katA*::(*loxP-t_rrnB_-P_tacA_-mCherry-t_T7_*), Δ*mptG,* pCM410 | [10] |
|  |  |  |
| **Plasmids** |  |  |
| pCM410 | pCM160 bearing the *flhA*-*fghA* cassette downstream of *P_mxaF_* | [10] |
| pHC112 | pCM410 bearing *P_lac_-cymR* and a CymR binding site (*CuO_cmt_*) between *P_mxaF_* *and the flhA*-*fghA* cassette | [34] |
| ^b^pCM410^Index^ | Derivatives of pCM410 | This study |
| ^b^pHC112^Index^ | Derivatives of pCM112 | This study |
|  |  |  |

^a^Km^r^, kanamycin resistance

^b^Index, plasmid derivatives of pCM410, pCM160, pHC112 carrying mutations that occurred on pCM410 during experimental evolution of the EM strain. See Figure 1B and Table S1 for details.
