# Supplementary material for: Mapping the Fitness Landscape of Gene Expression Uncovers the Cause of Antagonism and Sign Epistasis between Adaptive Mutations
Source: PLoS Genet. 2014 Feb 27;10(2):e1004149. doi: 10.1371/journal.pgen.1004149 (PMC3937219; doi:10.1371/journal.pgen.1004149)
Supplement: Table S3 — The value of fitted parameters in the fitness landscape model. (DOCX) [file pgen.1004149.s006.docx]

**Table S3. The value of fitted parameters in the fitness landscape model.**

| **Parameter** | **Value** |
| --- | --- |
| *V*_max_ | 175.841 |
| E*_h_* | 0.186 |
| Threshold | 173.230 |
| *C*_1_ | 0.011 |
| *C*_2_ | 0.002 |
